# Perfume and Flavor Engineering: A Chemical Engineering Perspective

**DOI:** 10.3390/molecules26113095

**Published:** 2021-05-22

**Authors:** Alírio E. Rodrigues, Idelfonso Nogueira, Rui P. V. Faria

**Affiliations:** Laboratory of Separation and Reaction Engineering, LSRE-LCM, Department of Chemical Engineering, Faculty of Engineering, University of Porto, 4200-465 Porto, Portugal; idelfonso@fe.up.pt (I.N.); rui.faria@fe.up.pt (R.P.V.F.)

**Keywords:** perfume engineering, flavors and fragrances, perfumery ternary diagram, classification of perfumes, perfumery radar, trail of perfumes, perfume performance, evaporation and diffusion of perfumes, effect of matrix, flavor engineering

## Abstract

In the last two decades, scientific methodologies for the prediction of the design, performance and classification of fragrance mixtures have been developed at the Laboratory of Separation and Reaction Engineering. This review intends to give an overview of such developments. It all started with the question: what do we smell? The Perfumery Ternary Diagram enables us to determine the dominant odor for each perfume composition. Evaporation and 1D diffusion model is analyzed based on vapor-liquid equilibrium and Fick’s law for diffusion giving access to perfume performance parameters. The effect of matrix and skin is addressed and the trail of perfumes analyzed. Classification of perfumes with the perfumery radar is discussed. The methodology is extended to flavor and taste engineering. Finally, future research directions are suggested.

## 1. Looking Back: The Beginning of Perfume Engineering at LSRE. What Do We Smell?

In the late 1990s, research on perfume engineering at the Laboratory of Separation and Reaction Engineering (LSRE) started with one of our members (AER) and a posdoc student, Vera Mata. She was had PhD in porous media and wanted to be an entrepreneur. She was interested in perfumes and we wanted to answer, with engineering tools, the question: What do we smell? Can we predict it?

**Figure 1:**
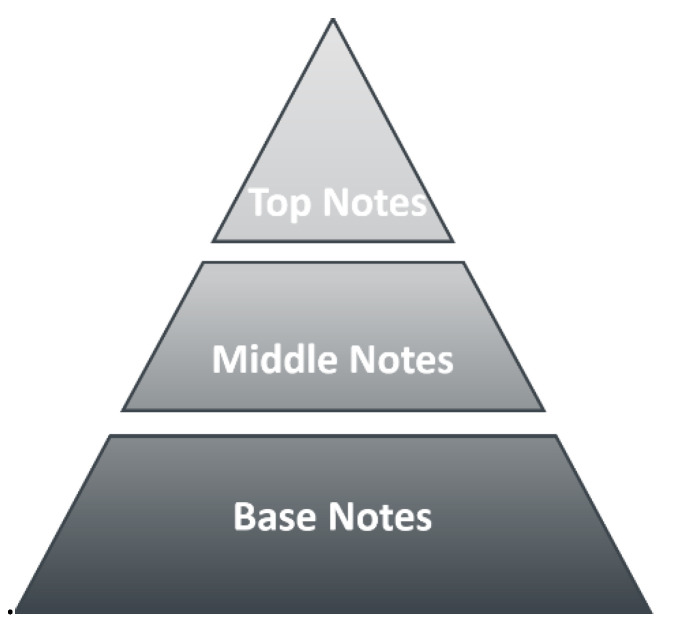
The structure of a perfume represented by Carles’ pyramid.

A perfume, according to Jean Carles [1], is a liquid mixture of top notes (first impact, fresh), middle notes (main perfume character) and base notes (long-lasting) in solvents (ethanol, water, matrix). The structure of a perfume is shown in Figure 1 as Carles’s pyramid.

It should be noted that perfumed products appear in our everyday lives as fine fragrances (Chanel No. 5), air care (candles), fabric care (detergents), personal care (shampoos), personal wash (bar soaps), home care (dish wash), etc.

The Flavors and Fragrances (F&F) industry has a large palette of essential oils and fragrances (~10^4^) to formulate products, mainly developed by perfumers. At the time we started research in this area, formulation by trial and error (~1000 tests) implied long development times (1–3 years) and production costs (perfumery raw materials, PRM, can cost from $10 up to $50,000 per kg). The F&F industry involved businesses of $26 bn in 2017 [2]. The market share is around 33% in the USA, 28% in Western Europe, 28% in Japan and China and 11% in the rest of the World. More than 78% of the market in 2017 was shared by the top 10: Givaudan, Firmenich, IFF, Symrise, Mane SA, Frutarom, Takasago, Sensient Flavors, Robertet SA, T. Hasegawa.

Perfume engineering is a branch of product engineering that caters to consumer needs for a specific application or market by providing a new valuable product, following the path: needs, ideas, selection and manufacturing [3]. The product classification of Raquel Costa et al. [4] is: commodities, specialty chemicals, formulated products, devices, virtual chemical products, bio-based products and technology-based consumer goods. Perfumes can be easily recognized as formulated products. Product engineering appeared with the shift in the evolution of the chemical industry from bulk chemicals to specific added-value products (electronics, flavors, coatings, fragrances, etc.).

**Figure 2:**
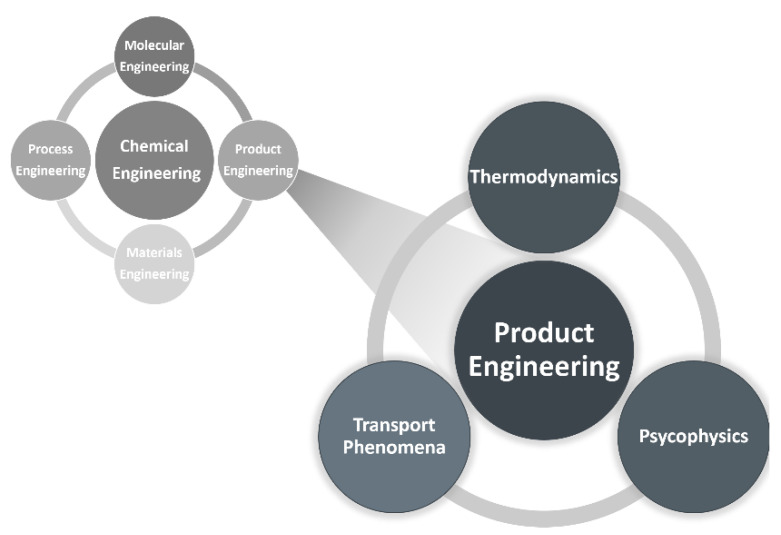
Chemical engineering today: ChE = M^2^P^2^E [5] and perfume engineering as a branch of product engineering [6].

Perfume engineering involves disciplines of thermodynamics, transport phenomena and psychophysics (Figure 2) to predict the odor of mixtures of fragrances, evaporation/release of fragrances, diffusion and performance of perfumes, as well as to predict odor detection thresholds and classify perfumes into olfactory families (perfumery radar).

## 2. The Perception of Odors

The pyramid structure of a perfume mentioned above considers top notes as those giving the initial impact of fragrance; typically citrus, with green notes lasting 15–30 min on the skin. The middle notes are typically spicy, leather, or floral, giving the perfume character (body), and lasting 3–4 h on the skin. Base notes are typically amber or musk, giving the substantivity of the fragrance, and lasting more than 4 h on the skin. These notes, together with solvents (ethanol, water, matrix), stabilizers, colorants, and UV filters, constitute the perfume.

The odor perception, from vapor to the nose, was addressed by Richard Axel [7] and Linda B. Buck [8], who won the Nobel Prize in Physiology or Medicine in 2004 “for their discoveries of odorant receptor and the organization of the olfactory system”. Odorants in the air bind to odorant receptors; the odorant receptor cells in the nasal epithelium are activated and send electric signals, which are relayed in glomeruli of the olfactory bulb and transmitted to higher regions of the brain.

The process for the perception of a perfume can be divided into four steps: first the **evaporation** of the perfume, followed by **diffusion** in the air until the olfactory system is reached, where there is the perception of **odor intensity** and **odor character**. The first two steps are the domain of chemical engineering and the last two belong to psychophysics.

### 2.1. Odor Thresholds

**Figure 3:**
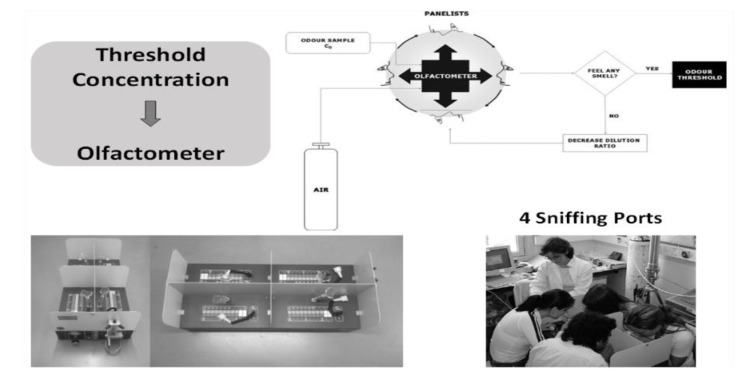
Measurement of ODT by a panel using an olfactometer at LSRE.

The Odor Detection Threshold (ODT) is the minimum concentration of an odorant that can be detected by humans, or, according to ASTM (method E 679-91), is the concentration of an odorous chemical at which the physiological effect elicits a response for 50% of the panelists. Human ODT in the air can vary by order of magnitudes from 0.3 ppb for t-butyl mercaptan, 40 ppb for menthol, 870 ppb for formaldehyde, 15,000 ppb for acetone and 141,000 ppb for methanol [9,10,11]. Recalling a story of a Ph.D. student working on sweetening gasoline and using t-butyl mercaptan in our lab; when getting on the crowded bus to go home at rush hour, a void space was nevertheless created around the student! The Odor Recognition Threshold (ORT) is the lowest odorant concentration at which its recognition becomes possible. ODT can be measured in the laboratory by a panel using an olfactometer as shown in Figure 3.

### 2.2. Odor Intensity Models

The simplest way to quantify the smell of a fragrance molecule *i* is the Odor Value (*OV_i_*) [12] defined as the ratio of its concentration in the gas-phase $C_{i}^{g}$ and its odor detection threshold *ODT_i_*, i.e.,

(1) $$OV_{i}=\frac{C_{i}^{g}}{ODT_{i}}.$$

A more realistic measure of the smell which, somehow, considers the saturation of the sensor (nose) is the odor intensity $\psi_{i}$ defined, according to Stevens Power Law [13], as

(2) $$\psi_{i}={\left(\frac{C_{i}^{g}}{ODT_{i}}\right)}^{n_{i}},$$

where *n_i_* is the power law exponent.

### 2.3. Odor Character Model for a Mixture—The Strongest Component Model

The simplest model to quantify the smell from a multicomponent mixture is to say that we smell the component with the higher odor value or higher intensity, i.e.,

(3) $$\begin{array}{l} OV_{mixture}=\max\{OV_{i}\}\\ \psi_{mixture}=\max\{\psi_{i}\} \end{array}$$

This is the idea behind the Strongest Component Model (SCM).

### 2.4. Sensory Dose/Response Curve

**Figure 4:**
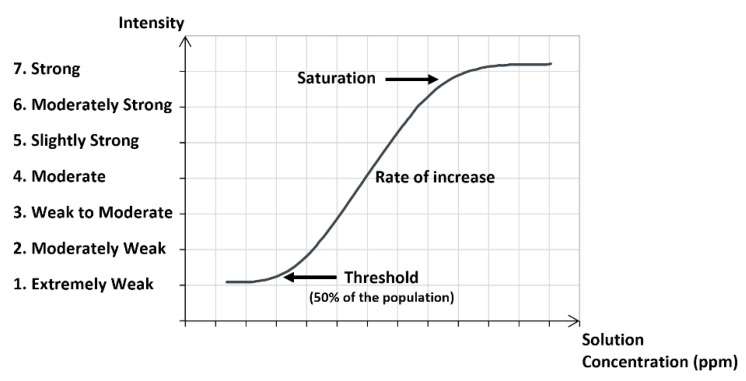
Sensory dose/response curve with a scale of odor intensity.

The sensory dose/response curve is a qualitative measure by panel members of the odor intensity for different concentrations in the liquid mixture. The scale of intensity goes from extremely weak (1) to strong (7). Figure 4 shows a dose/response curve indicating the threshold and the saturation.

**Figure 5:**
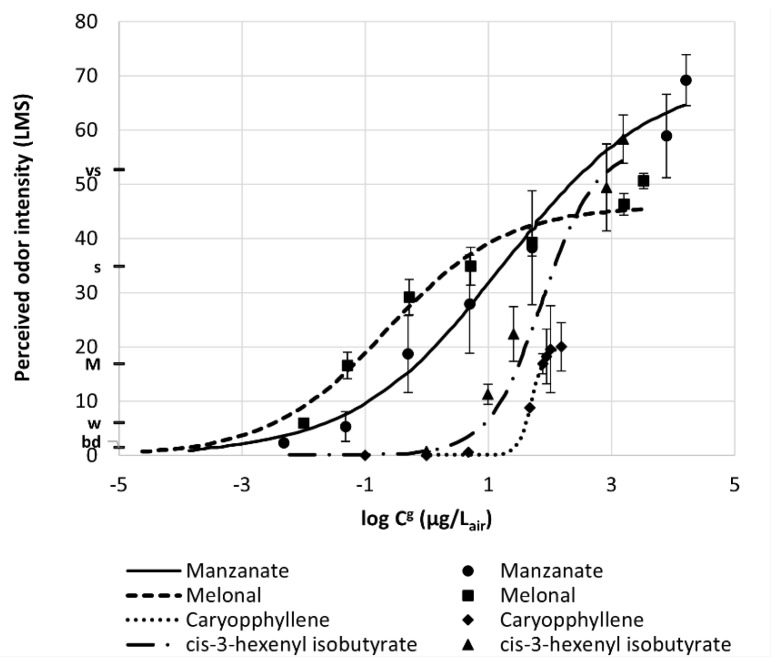
Odor Intensity Standard Curves in LMS scale versus the logarithm of fragrance concentration in air (Reprinted with permission from *Ind. Eng. Chem. Res*. 2019, 58, 15036−15044. Copyright 2019, American Chemical Society).

Researchers from Kao Corporation [14] developed a database for 314 perfumery raw materials (PRM) with Odor Intensity Standard Curves (OISC) showing the relationship between PRM odor intensity and gas concentration. They used the Labeled Magnitude Scale (LMS) of Green [15,16] with a range 0–100 with a quasi-logarithmic scale between labels: barely detectable (bd), 1.4; weak (w), 6.1; moderate (M), 17.2; strong (s), 35.4; very strong (vs), 53.3; and strongest imaginable, 100. Figure 5 shows OISC for manznate, melonal, caryophyllene and cis-3-hexenyl isobutyrate. This treatment is an extension of our methodology in the sense that it links values of odor intensity to the sensory evaluation of panelists.

### 2.5. Evaporation of Perfumes: Modeling Vapor-Liquid Equilibrium (VLE)

The first step to get the concentration of the fragrance in the gas-phase $C_{i}^{g}$ is to relate the liquid composition given by the mole fraction *x_i_* with the partial pressure of component *i* in the gas-phase *P_i_* = *y_i_P* with the Raoult’s law:

(4) $$y_{i}P=x_{i}\gamma_{i}P_{i}^{sat},$$

where $y_{i}$ is the mole fraction of species *i* in the gas phase, *P* is the pressure, $P_{i}^{sat}$ is the vapor pressure of species *i* and $\gamma_{i}$ is the activity coefficient of species *i* in the liquid phase. Taking into account the ideal gas law, the concentration of component *i* in the gas phase is

(5) $$C_{i}^{g}=\frac{x_{i}\gamma_{i}P_{i}^{sat}}{RT},$$

and its odor value is:

(6) $$OV_{i}=\gamma_{i}x_{i}\left(\frac{P_{i}^{sat}M_{i}}{ODT_{i}}\right)\left(\frac{1}{RT}\right),$$

where *M_i_* is the molecular weight. The key point is the prediction of vapor composition using group-contribution methods (UNIFAC, UNIFAC-D, ASOG, A-UNIFAC, COSMO-SAC) for calculating the activity coefficients [17,18]. The prediction of the sensorially-dominant odor was compared with the evaluation of perfumers with a correlation r^2^ = 0.806 [19].

### 2.6. Prediction of Odor Detection Threshold (ODT)

**Figure 6:**
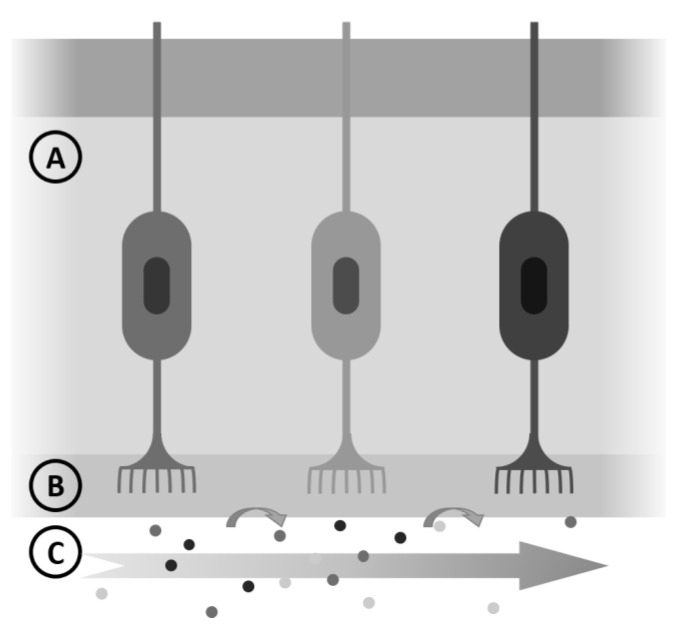
Simplified steps in odor detection: air and odorant molecules (C), mucus (B) and nasal epithelium (A).

The task of predicting ODT is not easy, but a simplified vision of the process of odor perception will be helpful. We will consider that odorant molecules evaporate and reach our nose ($P_{i}^{sat}$), dissolve in the mucus layer ($C_{i,W}$) and bind to specific olfactive receptors (OR) located in the cilia ($K_{OW}$); above a certain concentration (ODT) are detected. A sketch, as shown in Figure 6, aids in understanding.

Introducing partition equilibrium between the concentration of odorant in air and mucus (assumed as water) $K_{air/mucus}\propto K_{air/water}=\frac{P_{i}^{sat}}{C_{i,W}RT}$ and equilibrium between the odorant concentrations in the mucus and bio-phase (assumed as octanol) $K_{mucus/biophase}\propto K_{octanol,water}$ the ODT becomes:

(7) $$ODT_{i}\propto \frac{K_{air,W}}{K_{OW}}=\frac{P_{i}^{sat}}{K_{OW}C_{i,W}RT}.$$

Data from literature for 121 odorant molecules were correlated by

(8) $$log(ODT_{i})=(0.97\pm 0.05)log(P_{i}^{sat}/K_{OW}C_{i,W}RT)+(4.2\pm 0.2),$$

with a regression factor od r^2^ = 0.77 [20].

## 3. Perfumery Ternary Diagram (PTD)

**Figure 7:**
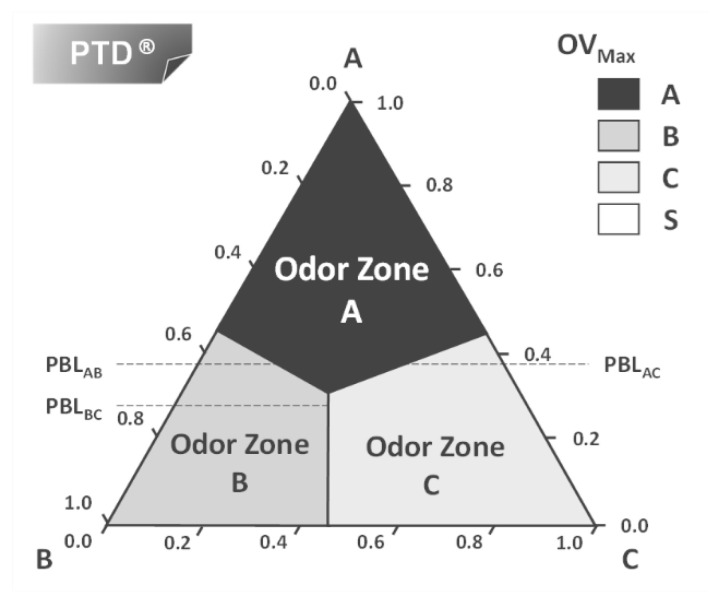
The perfumery ternary diagram: combining perfume pyramid structure with the ternary phase diagram.

An engineering tool to predict the smell of a liquid mixture was first presented by Mata and Rodrigues [21,22,23,24] and can be illustrated for the simple case of a mixture of one top note (A), one middle note (B) and one base note (C) in a solvent ethanol (S). The idea comes from engineering ternary diagrams using mixture compositions on a solvent-free basis and working with odor values (or intensity) for each component A, B, and C. For each liquid composition, the odor values (or intensity) are calculated and using the Strongest Component Model we can map the triangle in regions with different dominant odors. This is the Perfumery Ternary Diagram (PTD), shown in Figure 7. The distance from a point in the triangle to the AB line is the composition of C on a solvent-free basis $x_{C}^{\prime }=\frac{x_{C}}{x_{A}+x_{B}+x_{C}}$ where *x_A_*, *x_B_* and *x_C_* are the compositions in the whole perfume mixture. Similarly, the distance from a point to AC gives the composition of B and the distance to the line BC gives the composition of A. The lines separating odor zones are the Perfumery Binary Lines (PBL) where the OV of the two adjacent components are equal.

The idea was extended to quaternary (PQD) and quinary mixtures (PQ2D) by Teixeira [25] to allow visualization, but can be applied to a mixture of any number of components. What is needed is just:

(i) a database of vapor pressure for perfumery raw materials (PRM);
(ii) a database of ODT for PRM and
(iii) a tool to calculate activity coefficients for PRM.

**Figure 8:**
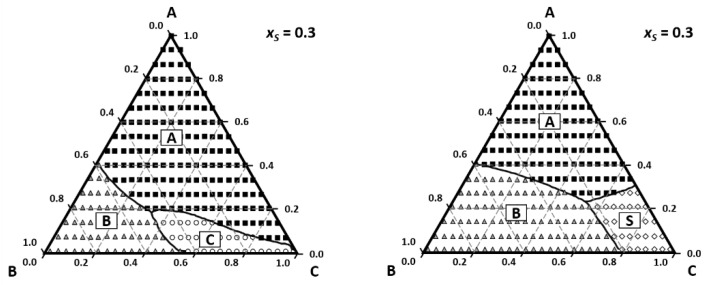
The effect of base note on odor zones on odor zones: **left** A—limonene (squares); B—geraniol (triangles); C—vanillin (circles); S—ethanol (losange); **right**—the base note C tonalide is not perceived and ethanol is (Reprinted with permission from AIChEJ, 2009, 55, 15. John Wiley and Sons).

The PTD tool allows us to analyze the effect of non-ideality of perfume mixtures on the odor zones as well as the description of smell with odor value or intensity from Steven′s law. The effect of base notes and fixatives can be easily visualized as illustrated in Figure 8. By changing the base note from vanillin (left) to tonalide (right) there is no region where we smell tonalide; instead, we smell ethanol.

## 4. Diffusion and Performance of Perfumes

### 4.1. Perfume Performance

**Figure 9:**
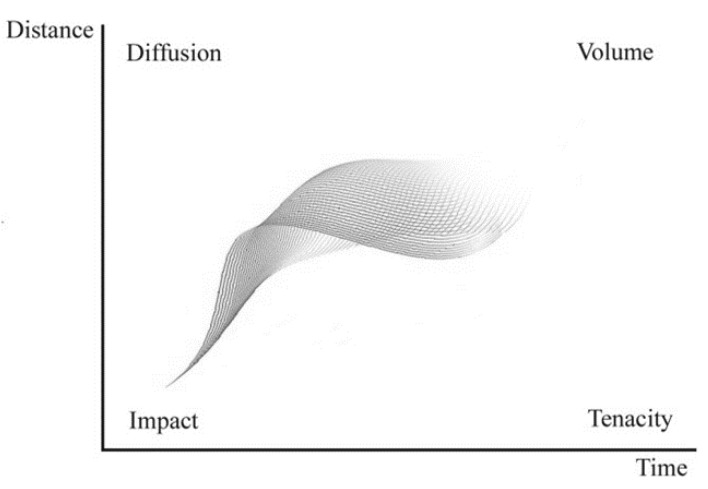
Perfume performance parameters (Reprinted with permission from AIChEJ, 2013, 59, 15. John Wiley and Sons).

In perfumery, the performance of a perfume is evaluated using the words “**impact**” (immediate olfactory odor sensation), “**tenacity**” (persistence of fragrance after some time near the source), “**diffusion**” (efficacy of perfume at some distance from the source) and “**volume**” (effectiveness of a perfume over time and distance). In chemical engineering terms, all we need is a model allowing the calculation of the concentration of perfume components as a function of time and distance, $C_{i}(z,t)$, and then converting that information into odor value or intensity. This is shown in Figure 9 from Teixeira [26].

**Figure 10:**
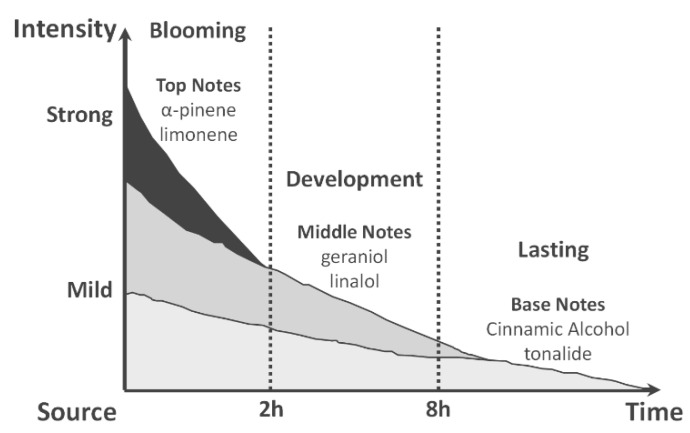
Blooming, development and lasting phases.

The top notes will be perceived first (blooming phase) followed by the middle notes (development phase) and then base notes will eventually show up in the lasting phase as sketched in Figure 10.

### 4.2. A Simple 1D Diffusion Model

**Figure 11:**
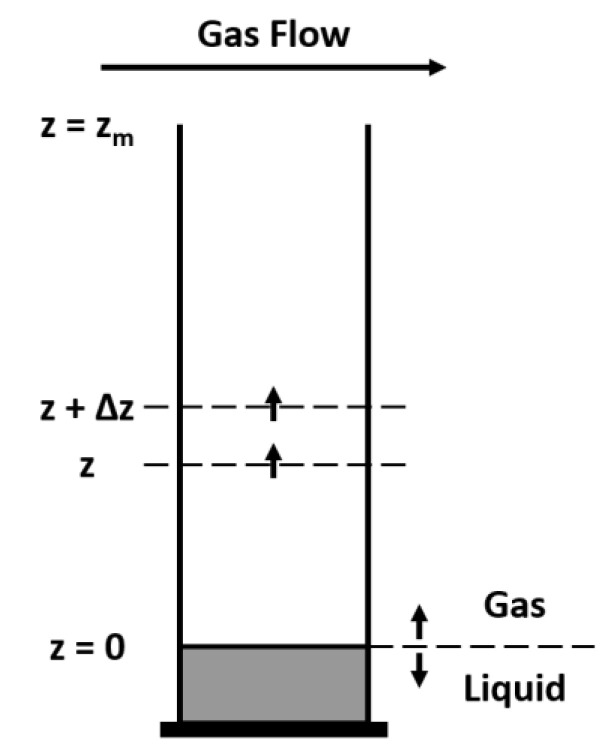
Diffusion tube with volume element of thickness Δz.

A 1D perfume diffusion model [27] is developed for the gas–liquid system involving a mass balance in a volume element of the gas phase with thickness Δz (Figure 11)

(9) $$\frac{\partial y_{i}}{\partial t}=D_{i,air}\frac{\partial^{2}y_{i}}{\partial z^{2}},$$

with initial condition $y_{i}=y_{i0}=0$ and boundary conditions at *t* > 0

(10) $$y_{i}=\frac{\gamma_{i}P_{i}^{sat}}{P}x_{i}=\frac{\gamma_{i}P_{i}^{sat}}{P}\frac{n_{i}}{\sum_{i}n_{i}}\;\mathrm{for}\;z=z_{0}$$

(11) $$y_{i}=0\;\mathrm{for}\;z=z_{m}.$$

In the above equations, *t* is the time variable, *z* is the axial space coordinate, *D_i,air_* is the diffusivity of species *i* in the air. Equation (10) represents the equilibrium at the gas-liquid interface. For the liquid phase the mass balance is:

(12) $$\frac{dn_{i}}{dt}=D_{i,air}A_{lg}{\frac{\partial y_{i}}{\partial z}|}_{z=0},$$

with initial condition $n_{i}=n_{i0}$ or $x_{i}=x_{i0}$, $x_{i}=x_{i0}$, where *n_i_* is the number of moles of species *i* in the liquid phase, and *A_lg_* is the area of the liquid/gas interface.

**Figure 12:**
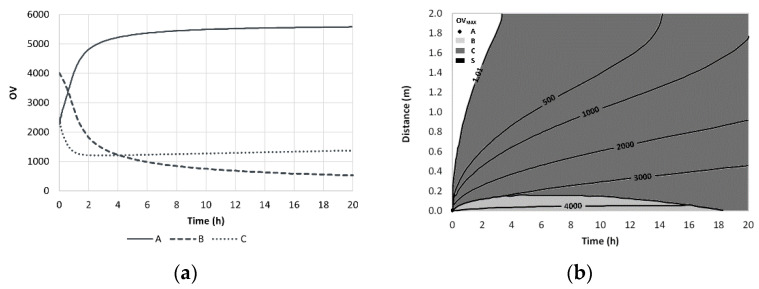
Time evolution of OV near the source- we smell limonene first and geraniol after 1 h (**a**) and iso-OV lines in a plot distance vs. time (**b**). Reprinted with permission from *Chem. Eng. Sci*. 2009, 64, 2570–2580, 2009, Elsevier.

The odor evolution near the source for a perfume mixture containing limonene (A), geraniol (B) and vanillin (C) is shown in Figure 12.

**Figure 13:**
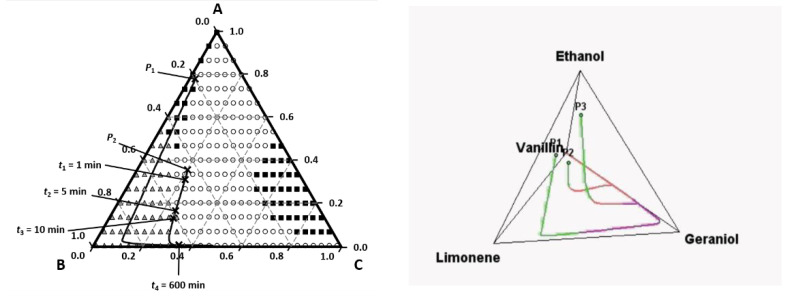
Evaporation lines of a perfume mixture near the source in PTD and PQD. Reprinted with permission from *Chem. Eng. Sci*. 2009, 64, 2570–2580, Elsevier.

Evaporation lines are shown in PTD and PQD diagrams at z = 0 (near the source) for a mixture of limonene (A), geraniol (B), vanillin (C) and ethanol (S). Depending on the initial perfume composition the smell follow different paths; for example, if we start with the mixture P1 we smell limonene first and then geraniol (Figure 13).

Diffusion models from a fixed source have been extended to account for the radial space dimension [28,29].

## 5. Perfume Classification and Perfumery Radar

Perfumery raw materials are classified in various categories by experts and are not usual for people. A classification of fragrances may have different layers related to emotions (culture, memories), look (color, texture), sensations (cool, dry, fatty, warm), aromas (floral, citrus, fruity, woody). There are classifications of fragrances, such as The Fragrance Wheel by Michael Edwards [30] or the Drom fragrance circle [31]. Most typical descriptors used in fragrance classification use a decreasing order in the percentage in an in-house database of 2000 odorants: floral, rose, diffusive, fruit, violet, green, musk, woody, herbaceous, citrus, spice, jasmine, amber, honey, liquor, marine, leather moss, tobacco. Examples of databases are those of Brechbill [32], Surburg and Panten [33], The Good Scents Company [34] and AIHA [35]. The 19 descriptors were used to choose eight olfactory families to be represented in a radar plot—the perfumery radar (PR)—to reduce the arbitrariness of perfume classification. The Perfumery Radar (PR) methodology [36] involves several steps:

(i) Classification of pure fragrances in *j* = 8 olfactory families: citrus, fruity, floral, green, herbaceous, musk, oriental, woody;
(ii) prediction of the odor intensity for each fragrance *i*, OV_i_;
(iii) calculation of the odor value for each family $OV_{j}=\sum_{i=1}^{N}w_{i}^{j}OV_{i}$, and normalization $OV_{j}^{\prime }=\frac{OV_{j}}{\sum_{j=1}^{L}OV_{j}}$;
(iv) plotting the perfumery radar.

**Figure 14:**
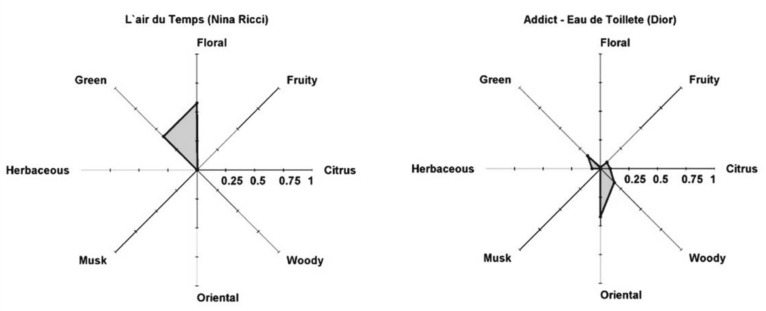
Perfumery radar of perfumes: **left**—L’ Air du temps (Nina Ricci); **right**—Addict-Eau de Toillete (Dior) (Reprinted (adapted) with permission from Perfumery radar: a predictive tool for perfume family classification, *Ind. Eng. Chem. Res.* 2010. Copyright 2010, American Chemical Society).

The weights $w_{i}^{j}$ presented are needed when one PRM is allocated to more than one family. The PR can be validated using GC-MS analysis of perfumes, family odor intensity model and comparison with headspace and perfume classifications. Examples of PR for feminine perfumes, such as L’Air du temps (Nina Ricci) and Addict-Eau de Toillete (Dior), are shown in Figure 14, together with company classification Floral and Floral–Green and Oriental and Oriental–Floral, respectively.

**Figure 15:**
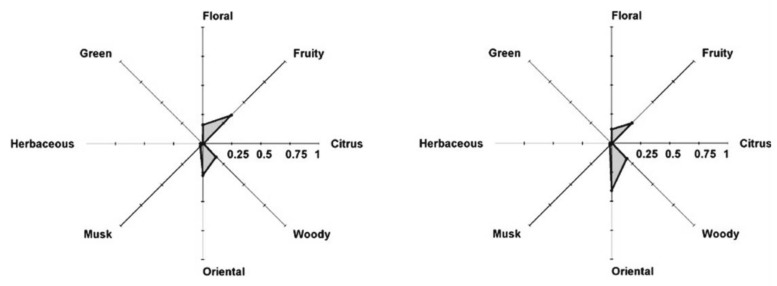
Perfumery radar of Gloria (Cacherel) at time *t* = 0 (**left**) and after *t* = 60 s (**right**). (Reprinted (adapted) with permission from Perfumery radar: a predictive tool for perfume family classification, *Ind. Eng. Chem. Res.*, 2010. Copyright 2010, American Chemical Society).

It is possible to combine the Perfumery Radar with a diffusion model and evaluate the evolution of perfume performance as shown in Figure 15 for Gloria (Cacherel) at *t* = 0 and *t* = 60 s. It is interesting to note that this perfume was classified by experts from various companies as oriental–woody (Osmoz and Fragrantica.com), oriental–fresh (Scent Direct), oriental–woody–floral (iPerfumer, Givaudan), floral–woody–amber (SFP), amber–rose (LT&TS), floral–oriental (Perfume Intelligence). The perfumery radar correctly predicts the olfactory families of several commercial perfumes. It is flexible to inserting new PRM in the database and uses scientific tools to predict the odor space instead of relying on the sensorial perception of people.

## 6. The Effect of Matrix and Skin

### 6.1. Effect of Matrix (Glycerine, Dipropylene Glycol, Skin Lotion)

It is expected that the odor of a liquid fragrance mixture will be influenced by the matrix in which it is present [37,38,39]: ethanol in a perfume, glycerine, dipropylene glycol in cosmetic matrices. The matrix effect was analyzed in our lab to study the release of *Origanum. majorana* L. from glycerine, dipropylene glycol and from a topical formulation –skin lotion. The supercritical fluid extraction with CO_2_ of the aerial part of *O. majorana* was carried out at 40 °C and 10 MPa. The extract was incorporated in the matrix in 0.01% (*v*/*v*). Dynamic headspace (DHS) coupled to GC-FID/MS was used to measure species concentration over time. The odor profile in the presence of glycerine was initially characterized by the fast release of β-caryophyllene, sabinene, p-cymene, limonene, myrcene, linalyl acetate and β-phellandrene followed by a high decrease in the next 5 h. The fast release is due to the hydrophobic nature of these compounds with low LogP_ow_, i.e., low affinity with the polar solvent glycerine. For example, the LogP_ow_ is 4.5 for limonene and 3.8 for linalyl acetate.

The odor intensity of the fragrance compounds was lower in dipropylene glycol (DPG) as a consequence of its high retention ability. Sabinene was the most released compound and after 2 h the headspace contained residual amounts of sabinene, myrcene, limonene, β-phellandrene, γ-terpinene and β-caryophyllene. DPG can be a good solvent to prolong the perceived scent of a fragranced product. In respect to the dipropylene glycol, the dominant odor changed as the time increased: the mixture started to smell like linalyl acetate (odor described as sweet, citrus, floral and woody) and then changed to myrcene (odor described as peppery, terpene, spicy and balsam). Despite the lower headspace amounts of myrcene compared to the remaining fragrance compounds, it will be more perceived due to its low ODT value (4.5 × 10^−2^ mg/m^3^).

When the matrix is a skin lotion, the initial headspace is reached in sabinene, myrcene, p-cymene, β-phellandrene and γ-terpinene and then a sharp decrease of concentration with time. The most retained components over time were linalyl acetate and β-caryophyllene but terpinen-4-ol increased after 5 h.

### 6.2. The Effect of Skin

**Figure 16:**
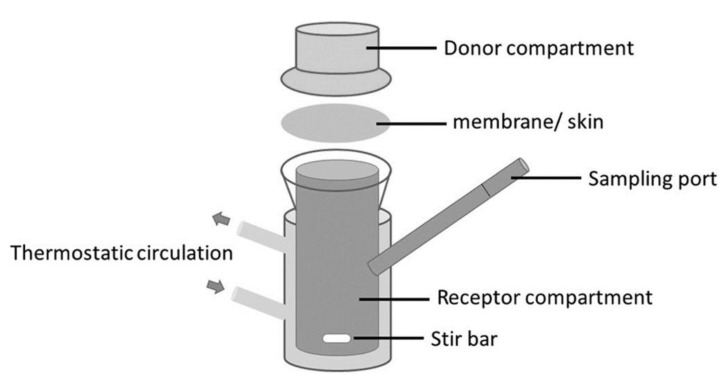
Franz cell for permeation studies of fragrance mixtures (Reprinted with permission from Elsevier, *Int. Journal of Biological Macromolecules* 2020, 147, 150–159).

The effect of the skin was studied in our lab with a Franz cell using skin prepared from pig ears according to the protocol described elsewhere [40]. The Franz cell shown in Figure 16 contains a donor compartment where the fragrance mixture is placed and a receptor compartment separated from the donor by the membrane (skin). It allows the study of the permeation of components through the skin and retention on the skin. At the same time, the odor in the gas phase in the donor compartment can be followed. The Franz cell is equivalent to the Wicke–Kallenbach diffusion cell known in Chemical Reaction Engineering [41].

**Figure 17:**
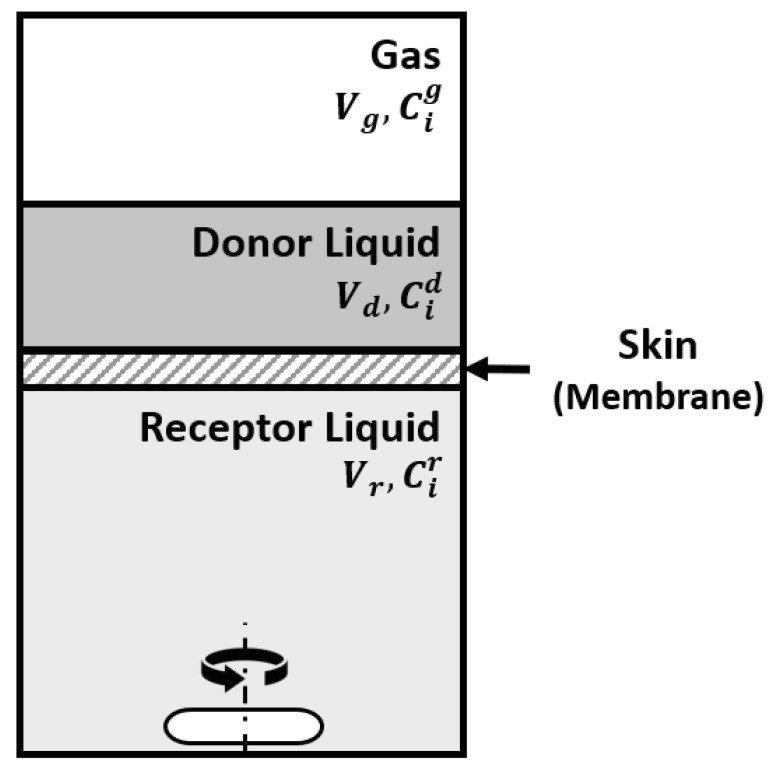
Sketch of the Franz cell system.

The modeling of the system sketched in Figure 17 involves a mass balance for the donor liquid solution

(13) $$-V_{d}\frac{dC_{i,d}}{dt}=AK_{p,i}(C_{i,d}-C_{i,r})-AD_{i,mix}{\frac{\partial C_{i}^{g}}{\partial z}|}_{z=0},$$

with the initial condition (t = 0) $C_{i,d}(0)=C_{i,d,0}$. In the above equation, *A* is the membrane area, $K_{p,i}$ is the permeation coefficient of component *i* through the skin, *V_d_* is the volume of the donor compartment, $C_{i,d}$ and $C_{i,r}$ are the concentrations in the donor and receptor compartments and $D_{i,mix}$ is the diffusivity of *i* in the gas phase above the liquid in the donor chamber.

The mass balance in the gas phase above the liquid in the donor compartment is:

(14) $$\frac{\partial C_{i}^{g}(z,t)}{\partial z}=D_{i,mix}\frac{\partial^{2}C_{i}^{g}}{\partial z^{2}},$$

with initial condition $C_{i}^{g}(z,0)=0$. The boundary conditions are:

(15) $$z=L,\;\frac{\partial C_{i}^{g}(L,t)}{\partial z}=0,$$

and at *z* = 0 the equilibrium at the gas–liquid interface

(16) $$C_{i}^{g}(0,t)=C_{i,eq}^{g}=\frac{M_{i}\gamma_{i}P_{i}^{sat}}{RT}x_{i}.$$

The mass balance for the receptor compartment is:

(17) $$V_{r}\frac{dC_{i,r}}{dt}=AK_{p.i}(C_{i,d}-C_{i,r})$$

**Figure 18:**
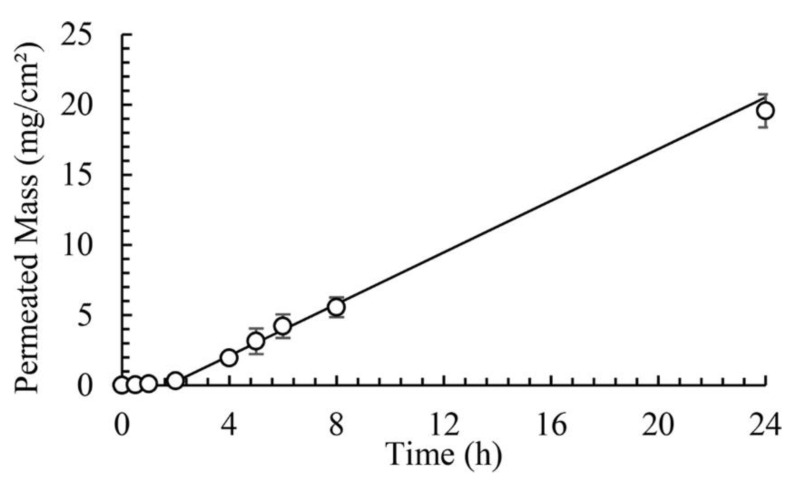
Cumulative amount of linalool in the receptor compartment versus time for the infinite-dose experiment (Reprinted (adapted) with permission from Evaporation and permeation of fragrance applied to the skin, *Ind. Eng. Chem. Res.,* 2019, 58, 9644–9650. Copyright 2019, American Chemical Society).

The permeation coefficients calculated from infinite-dose experiments were 1.08 × 10^−5^ cm/h, 8.25 × 10^−6^ cm/h and 2.15 × 10^−3^ cm/h for α-pinene, limonene and linalool, respectively. In all experiments, the fragrances were diluted in ethanol. This is illustrated in Figure 18 for the linalool experiment, infinite-dose.

The model allowed at the same time to follow the concentration in the headspace above the donor solution. Ultimately it would be important to separate the adsorption of each fragrance onto the skin. The release is affected by the interaction between fragrances and ethanol and also by the vapor pressure of the species. Vapour pressure and permeation coefficients in the skin were measured by Almeida et al. [42] for 14 PRM: camphor, carvacrol, carvone, citronellol, eucalyptol, eugenol, geraniol, limonene, linalool, menthol, menthone, tonalide, vanillin and α-pinene. Various models have been proposed to address dermal absorption of fragrances and drugs [43,44,45,46].

## 7. The Trail of Perfumes

The trail of perfumes or sillage is something we deal with in our everyday life. It describes the scented trail left by the fragrance wearer. It is determined by how long a fragrance travels away and diffuses around the wearer [47]. We tackled this problem by first analyzing the diffusion of fragrances released from a moving source [48]. First, we started with a 1D model considering molecular diffusion of a fragrance molecule (α-pinene) in the air as the only mass transport mechanism. Considering an impermeable boundary condition and constant release of the fragrance $\mu_{i}^{mass}=k_{i}M_{i}C_{i}^{g}$ the gas concentration in a semi-infinite medium is:

(18) $$C_{i}(z,t)=\mu_{i}^{mass}\int_{t_{i}}^{t}\frac{1}{2{\left[\pi D_{i}(t-\tau )\right]}^{1/2}}\{exp-\frac{{\left(z-z_{0}(\tau )\right)}^{2}}{4D_{i}(t-\tau )}+exp-\frac{{\left(z+z_{0}(\tau )\right)}^{2}}{4D_{i}(t-\tau )}\}d\tau$$

**Figure 19:**
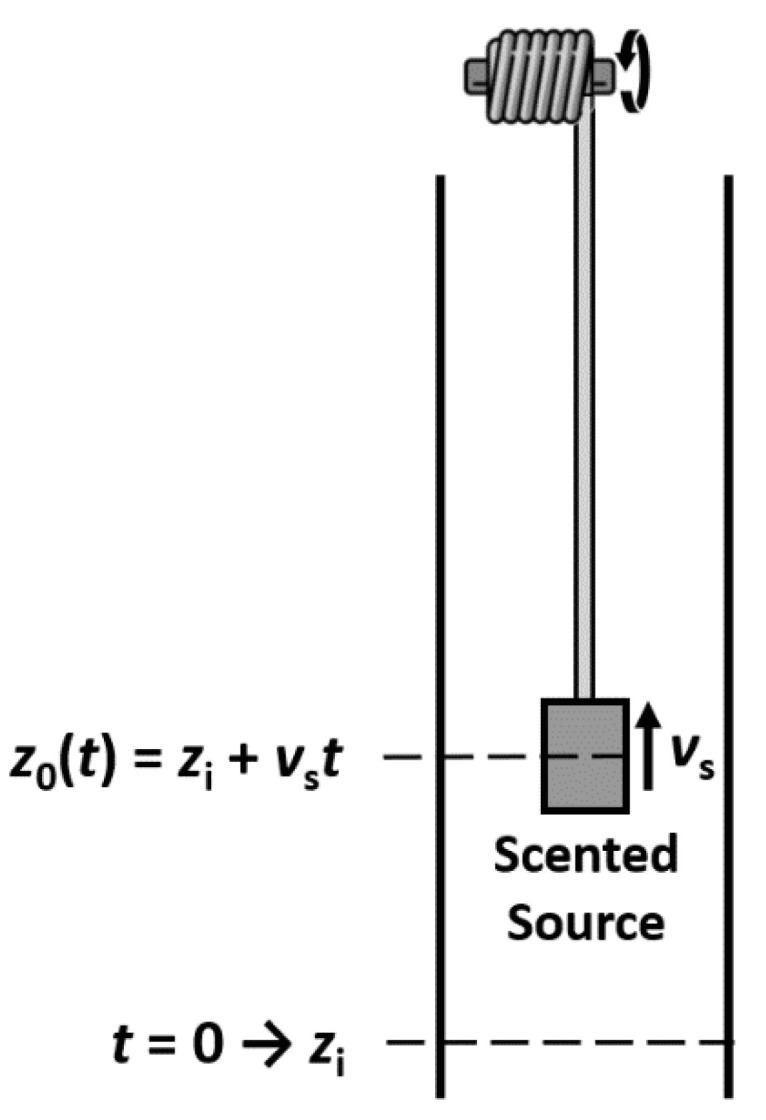
Diffusion tube and moving source.

**Figure 20:**
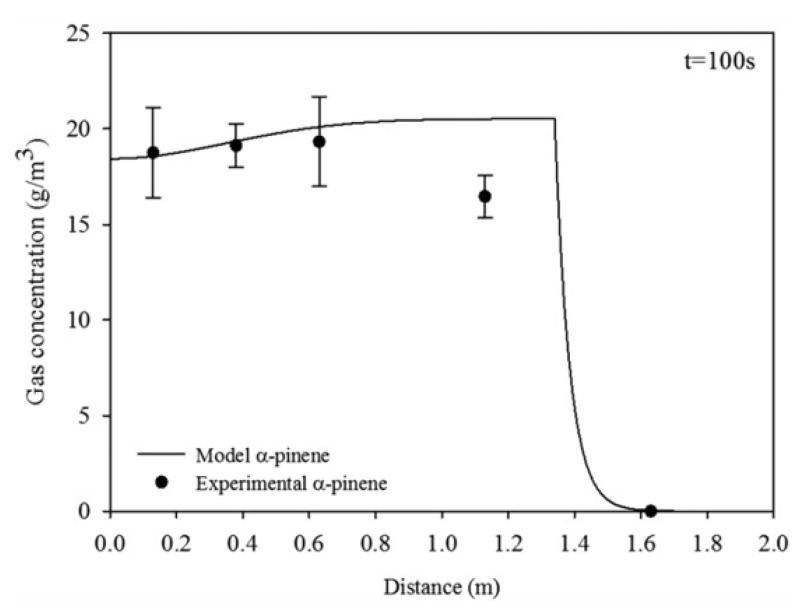
Simulated and experimental gas concentration profiles of a-pinene *versus* distance at t = 100 s of a source moving at 1.34 × 10^−2^ m/s and Dα-pin = 6.04 × 10^−6^ m^2^/s (Reprinted with permission from AIChEJ 2018, 64, 2890–2897, John Wiley and Sons).

The mass evaporation rate can be calculated as

(19) $$\mu_{i}^{mass}=k_{i}M_{i}C_{i}^{g}$$

where the mass transfer coefficient *k_i_* takes into account film contributions from gas and liquid sides. The validation was performed in a diffusion tube, and a system was developed to move the scented source along the axial direction (Figure 19). Results showed that experimental data fitted well with the numerical simulation, suggesting this model as a valid tool to describe the trail of a fragrance released from a moving source for low Reynolds number of the order of 10 (Figure 20).

In the case of a person walking at the speed of 1.34 m/s in a room or corridor inside a building, 3D models are required and mass transport of the perfume to the surrounding air will be dominated by turbulent diffusion or eddy diffusion Dt which is two orders of magnitude higher than molecular diffusion. For the 3D case the solution is:

(20) $$C_{i}(z,t)=\mu_{i}^{mass}\int_{t_{i}}^{t}\frac{1}{8{\left[\pi D_{i}(t-\tau )\right]}^{1/2}}\{exp-\frac{{\left(r-r_{0}(\tau )\right)}^{2}}{4D_{i}(t-\tau )}+exp-\frac{{\left(r-r_{1}(\tau )\right)}^{2}}{4D_{i}(t-\tau )}\}d\tau$$

where the source path is $r_{0}(t)={\left[x_{0}(t),y_{0}(t),z_{0}(t)\right]}^{T}$, $r_{0}(t)={\left[x_{0}(t),y_{0}(t),z_{0}(t)\right]}^{T}$ and $r_{1}(t)={\left[x_{0}(t),y_{0}(t),-z_{0}(t)\right]}^{T}$.

**Figure 21:**
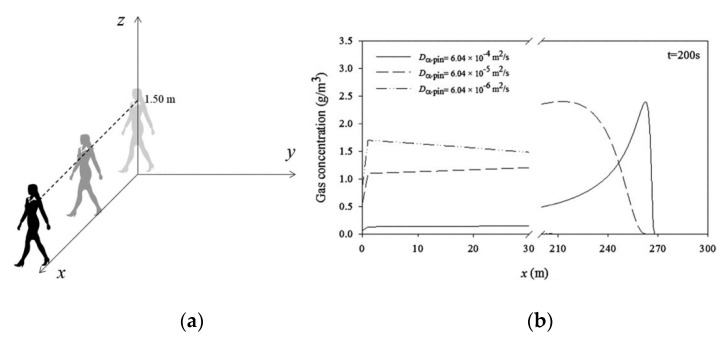
3D model- moving source at 1.50 m (**a**) and concentration profiles for 3 values of diffusivity evaluated at *z* = 1.60 m and *t* = 200 s (**b**) (Reprinted with permission from AIChEJ 2018, 64, 2890–2897. John Wiley and Sons).

As an example for an initial position of the moving source **r**_0_ (0, 0, 1.50 m) is shown in Figure 21a, and the concentration profiles at *t* = 200 s for a person with a fragrance α-pinene moving at 1.34 m/s are shown in Figure 21b.

These models can help the fragrance industry to achieve the desired trail of fragrance more quickly and efficiently. The search for devices which can be used to measure the sillage of fragranced products continues [49].

## 8. Flavor Engineering

The methodology developed for perfume engineering can be extended to taste engineering or flavor engineering. We previously defined the odor detection threshold as the minimum gas phase concentration at which an odorant is detected by the nose; and the odor value OV as the ratio between the concentration of odorant in gas-phase divided by the ODT. Similarly, we define the flavor detection threshold FDT_i_ as the lowest liquid concentration of component *i* at which it is detected by the retronasal route; the flavor value FV_i_ is then the ratio between the gas phase concentration and its FDT.

**Figure 22:**
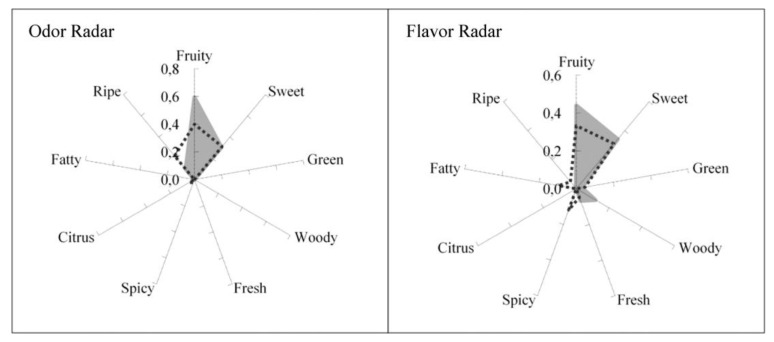
Odor and flavor radars for peach juice (experimental; shaded area–predicted). Reprinted with permission from *Ind. Eng. Chem. Res.* 2018, 57, 8115−8123 Copyright 2018, American Chemical Society).

The idea is to predict the sensory quality of flavored products based on their gas phase composition with the help of psychophysical models and olfactory descriptors. The first tested case of flavored products was fruit juices (peach, lemon, mango and pineapple). For each fruit juice, the headspace gas phase composition was measured by chromatography. The tests were extended to binary and ternary mixtures of fruit juices [50]. Odor and flavor radars were constructed with families of fruity, sweet, green, woody, fresh, spicy, citrus, fatty, ripe tones and validated by a sensory evaluation of consumers as shown in Figure 22 for peach juice. It should be noted that the headspace of peach juice contained various compounds identified as ethyl butyrate (fruity, sweet, spicy) isoamyl acetate (sweet, fruity, ripe), benzaldehyde (woody, fruity, sweet), ethyl hexanoate (sweet, fruity, green), limonene (citrus), linalool (citrus, sweet, woody). When one component is allocated to just one family, the weight is 1; when allocated to three families the weights are 0.6, 0.3 and 0.1. The ODTs for the six components are 3.35 × 10^−4^ mg/m^3^ for ethyl butyrate, 4.99 × 10^−1^ for isoamyl acetate, 6 for benzaldehyde, 1.5 × 10^−2^ for ethyl hexanate, 6.19 × 10^−1^ for limonene and 9.33 × 10^−3^ for linalool. The flavor detection thresholds FDT_i_ (mg/kg) for the same six components are 1.8 × 10^−3^, 5.7 × 10^−2^, 5.3 × 10^−1^, 8.0 × 10^−3^, 2.1 × 10^−1^ and 3.3 × 10^−3^, respectively.

A review on the biotechnological production of non-volatile flavors has been published recently by Paulino et al. [51]. Methodologies to advance the understanding of flavor chemistry have been proposed by Menis-Henrique [52] and the development of a model mouth discussed in detail by Panda et al. [53]. Encapsulation of flavors/aromas in food applications has been discussed by Gupta et al. [54].

## 9. Looking Ahead

**Figure 23:**
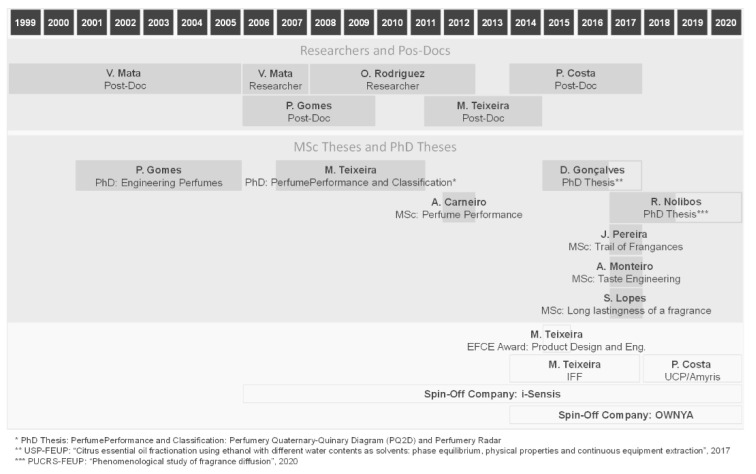
Contributors for research in Perfume Engineering started by Alírio Rodrigues and Vera Mata at LSRE.

More than two decades have past since research on perfume engineering started at LSRE. Figure 23 shows the timeline and the main contributors to the developments of perfume design: perfumery ternary diagram (PTD) and extension to quaternary and quinary systems (PQD and PQ2D), prediction of odor thresholds and VLE, performance of perfumes—1D and 3D diffusion models—and trail of perfumes, classification of perfumes—perfumery radar, effect of matrix and skin, extension to flavor/taste engineering. We have not listed many trainees coming from France, Poland, Spain or Brazil.

Looking ahead our research directions are focused on:

(i) The importance of diffusivity on the trail of perfumes or sillage

Brahms and Benaim [47] consider that in a perfume there are top notes and base notes and the heart (middle) notes come both from top and base notes molecules with fast diffusivity (sprinters) or slow diffusivity (long runners). This is an interesting view that requires a closer look at the measurements of diffusivities [55,56,57] and molecular design.

This reference to the importance of diffusivity goes back to the work of Mookerjee et al. [58,59] on the Aura of Aroma. The Aura of Aroma^®^ (AoA) technology consists of sampling the molecules from the air surrounding a liquid fragrance by Solid-Phase Micro-Extraction (SPME). The SPME needle should be as close as possible to the source, without ever touching it, and the sampling period is typically comprised between ½–1 h. In one of the first references to the AoA, the authors state that it is almost exclusively dependent on the species diffusivity, and independent from its “boiling point, molecular weight, odour threshold, or odour value”. Nevertheless, our previous studies suggest that VLE is expected to have an important role in the AoA. To test this hypothesis, the composition of the vapor phase in equilibrium with a reconstituted orchid (dendrobium superbum orchid) liquid fragrance was computed by applying modified Raoult’s Law and estimating the activation coefficients of all the species through the UNIFAC model (details can be found on the Supplementary Materials). The results were then compared with the composition of the AoA reported by Mookerjee et al. for this fragrance (Table 1).

As stated by Mookerjee et al., the composition of the AoA is considerably different from the composition of the liquid fragrance. For instance, the relative amounts of benzyl acetone, benzyl acetate linalool and 2-Tridecanone increase from the fragrance to the AoA (1.5, 26.0, 15.5 and 275 times, respectively). On the other hand, the relative amounts of oxyphenolon, 2-pentadecanone and ethyl myristate decrease (by 0.1, 0.5 and 0.6) from the liquid fragrance to the AoA. The gas phase composition values estimated from the VLE were slightly different, but respected the same general trends. The largest deviations relative to the AoA were observed for Linalool, 2-Tridecanone and 2-Pentadecanone. Through this rather simple approach, one is led to believe that, although VLE calculations alone do not describe the AoA accurately, they allow us to predict if the relative amount of a species in the gas phase is expected to increase or decrease relative to the liquid phase. Including the effect of the compound’s diffusivity would expectedly improve the preliminary estimations obtained through the VLE.

A closer look at this topic is required using tools to track perfume composition and sillage [60]. Related to this topic is the area of olfactive marketing where CFD tools are important.

(ii) Formulation of perfumes and fragranced products

The methodology of perfumery ternary diagram (PTD) was a pioneering idea that can be extended to fragrance mixtures of N components to find compositions delivering a certain smell. It can be further elaborated to include the effect of skin on the evaporation of perfumes. The Perfumery Radar can be extended to other areas (wines) and the methodology extended to taste/flavor engineering.

Zhao et al. [61] applied a model for the prediction of intensity and character of fragrances, across three main consumer touchpoints for the laundry process. They used headspace gas chromatography data with psychophysical models (Steven’s power law) and tested with a trained sensory panel. The authors claim it is the first time that the approach was validated under realistic conditions for a multi-component perfume mixture diluted in a structured product base. The formulation of fragrance products to be applied in detergents is described in the patent by Teixeira et al. [62] where specific terminology (experimental velocity—distance traveled 60 cm divided by the speed of diffusivity— time needed for olfactory detection at 60 cm of the mixing point) is used to classify fragrance ingredients.

Fragranced products include microcapsules of polyurethane-urea with perfume as a core material for textile applications such as perfumed suits [63] or eco-friendly microcapsules based on chitosan and Arabic gum [64], a research area at LSRE. Microencapsulation of fragrances is also a way of increasing the life of fragranced products [65].

It is important to say that the engineering tools developed in our group have some challenges to overcome: the prediction of the odor of chiral molecules and the prediction of the odor quality of a molecule, which may change with its concentration. However, having a database with the descriptors of enantiomers allow the use of the engineering tools presented, and VLE methods such as COSMO-SAC [18] may help in predicting the gas phase composition of chiral molecules from a liquid mixture.

It should be said that many odorant molecules are chiral. Leffingwell database collects more than 400 enantiomers and their odors; about 60% of the pairs have similar smells and 40% smell differently. One example is limonene: R-limonene smells orange and S-limonene smells lemon; another example is carvone: R-carvone smells mint and S-carvone smells like caraway. Several attempts have been made over the years to predict the smell of chiral molecules starting with the “shape” theory: “lock and key” between the odorant and receptor based on Pauling and Delbruck idea [66]. The shape alone does not explain reality, otherwise, virtually all pairs should smell different. Later, the pros and cons of the vibrational theory of Wright [67,68] and Dyson [69], later taken by Turin [70] and reviewed by Meierhenrich et al. [71] were also considered. Other researchers, such as Brookes et al., tried to correlate odor with molecular flexibility claiming that flexibility allows left and right-handed molecules to be distinguished [72].

Another point to consider is when the odor quality changes with odorant concentration as already reported by perfumers. One example is indole which smells floral at low concentrations and putrid at high concentrations. Tentative explanation pointed out shifts in the patterns of glomeruli activated by the odorant although other reports see no shift in location and simply increase in the number of glomeruli activated at low concentration of odorant [73]. Nevertheless, the engineering tools presented are still useful for predicting the odor from a liquid mixture if a database with descriptors of chiral molecules are available.

(iii) Artificial intelligence (AI) and perfume design

The advent of fast computing, data digitalization, cloud data storage systems, and several other tools from Industry 4.0 is changing several industrial paradigms. Among these technologies, Artificial Intelligence (AI) is presented as the possibility for machines imitating the intelligence and behavior of humans. AI is a key technology that can make use of the increasing big databases to extract useful information and disrupt the way that products are designed and developed. The concern about the potential of AI has also been an increasing issue in the literature [74]. Big-data is an issue that is becoming common in several sectors. Modern technology has allowed the generation and storage of a huge amount of data, which represents a potential to be explored.

The perfume industry is not an exception in this process, and it is possible to note a recent movement in this field towards the application of AI in perfume development. For instance, predicting the relationship between the structure of a molecule and its odor (quantitative structure-odor relationship, QSOR), is a difficult task [75,76]. One of the first reports in the direction is presented by Zhang, L. et al. [77], an AI model was trained to learn fragrance molecule classification, building a computer-aided molecular screening tool. The proposed model demonstrated a remarkable accuracy in performing the screening. Yu et al. [78] present a comprehensive review about the application of computer-based strategies in the design of experimental designs to flavor and sensory analysis. The referred work shows the potential of these techniques, including Artificial Neural Networks (ANN), for food flavor applications.

This topic has also caught the interest of multi-billionaire companies, such as ALPHABET. Sanchez-Lengeling et al. [79] developed graph neural networks, in ALPHABET’s subsidiary, Google, to quantify the relationship between molecular structure and odor (quantitative structure-odor relationship, QSOR).

AI is already in the fragrances business, the International Business Machines (IBM) working together with Symrise and O Boticário launched the project Phylira, to develop an AI-based tool to create perfumes using their big databases. The support from IBM on predicting natural language descriptions of mono-molecular odorants was published recently [80]. In this same line, Givaudan launched, in 2019, the project Carto, an AI-powered tool that brings science and technology to the development of new perfume formulation. It uses the proprietary ingredients ‘Odour Value Map’ to maximize the olfactive performance in the final formula.

It is interesting to mention the DREAM Olfaction Prediction Challenge-a crowd-sourced competition to develop models that can predict how a molecule smells from its physical and chemical features. Results from the DREAM Consortium were published recently in Science [81].

Most recently, a step toward a more complex application of AI in the Flavour and Fragrance field was presented by Zhang, X. et al. [82] and Santana et al. [83]. In Zhang’s work, the authors proposed a strategy to develop AI models to be applied in an optimization problem that searches to identify a fragrance formulation to deliver a certain odor. On the other hand, Santana’s work presents one of the firsts application reports of a more sophisticated AI tool, Deep Learning (DNN), to address dynamic-related problems found in the perfume formulation. The DNNs are a group of machine learning techniques that use types of more complex architectures of artificial neural networks to solve difficult problems. The authors proposed a framework that makes use of DNN models and meta-heuristic optimization (stochastic optimization algorithm) to systematically formulate fragrances, considering their behavior through time and space.

This research line driven by Idelfonso Nogueira (LSRE) aims in the future to build a novel smart Cyber–Physical System (CPS) for the on-demand design and production of perfumes. It will be based on current emerging technologies: systems automation, artificial intelligence and real-time optimization (RTO), and get them to work harmoniously in a CPS enabled by the Internet of Things (IoT).

## Figures and Tables

**Table 1:** Comparison between the Aura of Aroma^®^ measured for a reconstituted orchid (dendrobium superbum orchid) liquid fragrance and the gas-phase composition estimated from VLE.

Species	Oil (wt%)	Aura of Aroma^®^ (wt%)	VLE Gas-Phase Composition (wt%)
Benzyl acetone	0.02	0.03	0.17
Benzyl acetate	0.20	5.20	5.55
Linalool	2.20	34.10	57.05
Raspberry ketone	11.90	1.70	4.11
2-Tridecanone	0.02	5.50	0.04
2-Pentadecanone	69.00	33.50	25.62
Ethyl myristate	14.80	8.50	4.57

## Data Availability

Not applicable.

## Supplementary Materials

The following are available online, Table S1: Physical properties of the studied compounds; Table S2: UNIFAC sub-groups frequency table for the studied compounds.
