# Supplementary material for: Perfume and Flavor Engineering: A Chemical Engineering Perspective
Source: Molecules. 2021 May 22;26(11):3095. doi: 10.3390/molecules26113095 (PMC8196857; doi:10.3390/molecules26113095)
Supplement: Supplementary file 1 [file molecules-26-03095-s001.zip › molecules-1227639-supplementary.pdf]

molecules MDPI

Supporting information

Notes on the vapor-liquid equilibrium (VLE) estimations

In the present study, the vapor-liquid equilibrium was assumed to follow the Raoult's Law, expressed by:

$$y_i P = x_i \gamma_i P_i^{Sat}$$

where, x_i and y_i are the molar fraction of compound i in the liquid and gas phases, respectively, γ_i is the activity coefficient of compound i, and P and P_i^{Sat} are the total system pressure and compound i saturation pressure, respectively.

The physical properties of the compounds of interest are presented in the Table S1, including their saturation pressure. It is important to mention that, according to the data provided by Mookerjee et al.[1], the liquid phase composition of the reconstituted orchid (dendrobium superbum orchid) liquid fragrance is incomplete ($\sum_i^{NC} x_i^{exp} = 0.981$). As this problems deals with vapor-liquid equilibrium of an oil, the missing mass fraction was attributed to a mineral oil, hexadecane.

Table S1. Physical properties of the studied compounds.

Species	CAS	MW (g/mol)	Psat (Pa)	Tb (K)
Benzyl acetone	2550-26-7	148.2017	7.47	506.15
Benzyl acetate	140-11-4	150.18	21.9	485.15
Linalool	78-70-6	154.3	22.10	471.65
Raspberry ketone	5471-51-2	164.2011	0.13	473.15
2-Tridecanone	593-08-8	198.3449	1.60	536.2
2-Pentadecanone	2345-28-0	226.3981	0.11	567.2
Ethyl myristate	124-06-1	256.4241	0.12	568.2
Hexadecane	544-76-3	226.4	0.66	560.05

The UNIFAC group contribution method was used to compute the activity coefficients of each species. The group partition adopted is presented in Table S2.

Table S2. UNIFAC sub-groups frequency table for the studied compounds.

Species	Benzyl acetone	Benzyl acetate	Linalool	Raspberry ketone	2-Tridecanone	2-Pentadecanone	Ethyl myristate	Hexadecane
CH3	0	0	3	0	1	1	2	2
CH2	1	0	2	0	10	12	12	14
C	0	0	1	0	0	0	0	0
CH2=CH	0	0	1	0	0	0	0	0
CH=C	0	0	1	0	0	0	0	0
ACH	5	5	0	4	0	0	0	0
ACCH2	1	1	0	1	0	0	0	0
OH	0	0	1	0	0	0	0	0
ACOH	0	0	0	1	0	0	0	0
CH3CO	1	0	0	0	1	1	0	0
CH3COO	0	1	0	1	0	0	0	0
CH2COO	0	0	0	0	0	0	1	0

The molecular van der Waals volumes and surface areas were obtained from the open literature as well as the group's binary interaction parameters. The activity coefficients were computed through the classical UNIFAC model [2].

1. Aura of Aroma®:A Novel Technology to Study the Emission of Fragrance from the Skin, Braja D. Mookerjee", Suba M. Patel, Robert W. Trenkle and Richard A. Wilson, INTERNATIONAL FLAVORS & FRAGRANCES INC, 1997
2. Poling, B.E., J.M. Prausnitz, and J.P. O'Connell, Properties of Gases and Liquids (5th Edition). 2001, McGraw-Hill
